# Veterinarians’ Perceptions of Farm Animal Pain and Views on Pain Management: Two Cross-Sectional Scenario-Based Surveys of Cattle and Pig Practitioners in Poland

**DOI:** 10.3390/ani16203199

**Published:** 2026-10-12

**Authors:** Katarzyna Żarczyńska, Dawid Tobolski

**Affiliations:** 1 Department and Clinic of Internal Diseases, Faculty of Veterinary Medicine, University of Warmia and Mazury in Olsztyn, 10-719 Olsztyn, Poland; katarzyna.zarczynska@uwm.edu.pl; 2 Department of Large Animal Diseases and Clinic, Institute of Veterinary Medicine, Warsaw University of Life Sciences, 02-787 Warsaw, Poland

**Keywords:** farm animal pain, cattle, pigs, veterinarians, pain perception, pain management, analgesia, animal welfare, veterinary attitudes, cross-sectional survey

## Abstract

Two anonymous web-based surveys were administered to veterinarians working with cattle or pigs in Poland between March 2025 and February 2026 (101 responses per survey). Questionnaires assessed demographics, seven pain-related statements (5-point Likert scale), and pain ratings for clinical scenarios (0–10). Analyses used nonparametric tests with Benjamini–Hochberg multiplicity control and exploratory multivariable logistic regression. Agreement that pain mitigation improves recovery was nearly universal (cattle, 100/101; pigs, 95/101). In cattle, difficult calving, thermal disbudding, and castration were most often rated as severely painful (≥8/10; 88/101, 69/101, and 60/101, respectively). In pigs, limb fracture had a median rating of 10 and was rated ≥8/10 by 85/101 respondents, whereas tail-docking ratings varied widely. After multiplicity control, female cattle respondents more strongly endorsed insufficient analgesic options, whereas male pig respondents more strongly endorsed lower pain sensitivity in pigs than in humans. The latter belief correlated with lower mean pain ratings (ρ = −0.298). Among pig veterinarians, routine pain discussion was associated with self-reported knowledge (OR 2.11, 95% CI 1.13–3.93) and perceived limitations in analgesic availability (OR 2.24, 95% CI 1.15–4.37). These findings highlight veterinarians’ support for pain relief and identify areas for continuing education, communication with producers, and practical guidance on pain management.

## Simple Summary

Pain in farm animals is an important welfare concern, and veterinarians influence how pain is recognized and treated on farms. We conducted two anonymous online surveys of veterinarians working mainly with cattle or pigs in Poland. Each survey included 101 responses. Participants assessed statements about pain and rated the pain caused by common diseases and procedures on a scale from 0 to 10. Veterinarians in both groups strongly agreed that reducing pain improves recovery, although their views on available pain-relieving medicines and the severity of individual conditions varied. Difficult calving, thermal disbudding, and castration were commonly rated as severely painful in cattle, whereas limb fractures received the highest ratings in pigs. Ratings for tail docking in pigs varied widely. Female cattle veterinarians more strongly emphasized limited pain-relief options than male respondents. Male pig veterinarians more strongly agreed that pigs are less sensitive to pain than humans, and this view was associated with lower overall pain ratings. The findings identify areas for continuing veterinary education, communication with producers, and practical guidance on pain management.

## 1. Introduction

Worldwide, the growing importance of ethical considerations has increased public expectations regarding the welfare of farm animals. Freedom from pain is a central component of animal welfare; therefore, protection from pain, injury, and disease should rely primarily on prevention, prompt diagnosis, and effective treatment. Nevertheless, the development and implementation of methods for assessing and treating pain in farm animals remain less advanced than those for companion animals, for which numerous pain-assessment tools have been developed and validated [1,2]. Pain alters behavior, autonomic function, and neuroendocrine responses, thereby producing a negative affective state and representing a major cause of impaired animal welfare [3]. Nociceptive mechanisms are largely conserved among mammals, supporting the premise that farm animals can experience pain and require appropriate protection from its consequences [4]. Recognition that animals can experience pain and suffering underpins contemporary welfare frameworks, including the Five Freedoms and the Five Domains Model [5,6]. These frameworks emphasize the obligation to provide appropriate care and consider animals’ needs in human decision-making [3]. This principle is also reflected in animal-welfare legislation in many countries [7].

Cattle, sheep, and pigs may mask signs of pain, making clinical recognition difficult [8,9]. This does not imply lower sensitivity to pain. Because pain is subjective, no single measure unequivocally confirms its presence in another individual [3,10]. Physiological and neuroendocrine variables lack specificity because stress and fear can produce similar changes [10]. Behavioral indicators are therefore important, but interpretation requires knowledge of normal species-specific behavior [7].

In clinical practice, pain recognition depends primarily on the veterinarian’s experience and ability to interpret clinical signs. Pain perception is multifactorial and integrates behavioral changes, clinical examination findings, and knowledge of the disease course. Consequently, a veterinarian’s knowledge, experience, and individual beliefs may influence both the assessed severity of pain and the decision to initiate analgesic treatment [9,11]. Although veterinarians are expected to act in the best interests of animals, clinical decisions may also be shaped by personal ethical perspectives, willingness to critically evaluate established practices, and the degree to which those practices are accepted within a professional community [12]. Despite decades of research on pain physiology, assessment, and management, inadequate recognition and treatment of pain remain important problems in both human and veterinary medicine [13].

Previous studies have indicated that pain ratings and analgesic-treatment decisions may be influenced by veterinarian characteristics, including sex, professional experience, empathy, and type of practice [10,11,14,15,16,17,18]. Contemporary farm-animal practice increasingly applies herd health management principles [19]. Work organization, decision-making responsibilities, and relationships with producers differ across livestock sectors, supporting recognition of bovine and porcine health management as distinct areas of veterinary practice [20]. Such differences may affect exposure to painful conditions and opportunities for discussing pain management, but an organizational distinction alone does not establish a difference in pain perception.

To our knowledge, common pain-related attitudes have not previously been compared between cattle- and pig-focused veterinarians within Poland. This study aimed to describe and compare perceptions of pain and views on pain management in these two practice groups. We hypothesized that response patterns would be broadly similar for common attitudes and that differences would be more apparent for species-specific conditions and procedures. We also examined associations with sex, professional experience, and self-reported pain-related communication.

## 2. Materials and Methods

### 2.1. Study Design and Setting

A cross-sectional, anonymous, web-based survey study was conducted among practicing veterinarians working with cattle or pigs. Two parallel species-specific questionnaires shared seven attitudinal items and included species-relevant pain scenarios. Data were collected between March 2025 and February 2026, with timestamps recorded automatically by the survey platform. No change specifically to cattle- or pig-analgesia requirements in Poland was identified by the authors during this period.

### 2.2. Participants and Recruitment

Eligible participants were veterinarians actively engaged in field practice focused on cattle or pig medicine. Recruitment used purposive non-probability sampling through private e-mail correspondence and messaging or social platforms, targeting veterinarians known to work predominantly in one of these areas. Recruitment continued until a pragmatic target of approximately 101 responses per survey was reached. This target was not a basis for claiming population representativeness. No central sampling frame or reliable denominator stratified by predominant livestock practice was available, so neither a response rate nor a sampling fraction could be calculated. The questionnaire did not quantify the proportion of each respondent’s workload devoted to cattle or pigs. Because no identifiers linked questionnaires, participation of the same veterinarian in both surveys could not be excluded.

### 2.3. Survey Administration, Anonymity, and Data Handling

The questionnaire was administered using an online form platform. No personally identifying information, including names, e-mail addresses, or IP addresses, was collected or analyzed. All items were mandatory, so electronic item nonresponse did not occur. However, “I do not know” was a valid response and was treated as missing in analyses requiring numeric scores. Excluding such responses from Q4–Q9 produced complete-case regression samples of 98 cattle questionnaires and 93 pig questionnaires. Only fully completed questionnaires were included.

### 2.4. Questionnaire Structure

Each questionnaire comprised three sections. Demographic variables were sex, years in veterinary practice (1–5, 6–10, 11–19, or ≥20 years), and emergency medical responder or volunteer firefighter status. The latter variable was included on an exploratory basis because exposure to emergencies and human injury might be associated with perceptions of animal pain. It was not a validated measure of trauma exposure or empathy, and no directional hypothesis was specified. Respondents then rated seven pain-related statements from strongly disagree to strongly agree, with an additional “I do not know” option, and rated species-relevant clinical conditions and procedures on an 11-point numeric rating scale (NRS) from 0 (no pain) to 10 (most severe pain). The common items addressed pain recognition, self-perceived knowledge, relative pain sensitivity, perceived analgesic availability and cost, recovery benefit, and communication with producers. The original Polish wording and English translations are provided in Supplementary File S1 (Sections S1.1 and S1.2).

### 2.5. Pilot Testing

Before data collection, the Polish-language questionnaire was pilot tested in February 2025 by six individuals to assess clarity, ambiguity, and completion time. Minor editorial refinements were implemented, and pilot responses were excluded from the final dataset. The questionnaire was administered exclusively in Polish. The English translation was prepared solely for presentation in the manuscript and was not provided to participants. The pilot assessed comprehensibility and feasibility but did not by itself establish content validity or measurement validity.

### 2.6. Ethics

The study was non-interventional and anonymous, and participation was voluntary. Written introductory information described the purpose, planned scientific use and publication, anonymity, and approximate completion time before respondents chose whether to proceed. Proceeding with the questionnaire was treated as implied consent. No separate verbal consent procedure was used. The investigators considered formal ethics committee review unnecessary because the survey was anonymous, non-interventional and collected no sensitive personal data.

### 2.7. Statistical Analysis

The cattle and pig datasets were analyzed under an independent samples assumption. Analyses were performed in Python 3.14 using NumPy 2.5, pandas 3.0, SciPy 1.18, and statsmodels 0.15. Categorical variables were summarized as counts and percentages. Likert items were summarized using full and collapsed response distributions and medians with interquartile ranges (IQR). Responses were scored from 1 (strongly disagree) to 5 (strongly agree). “I do not know” responses were reported descriptively but excluded from numeric analyses.

Pain-intensity ratings (0–10) were summarized primarily as medians (IQR), with means and standard deviations (SD) as complementary measures. The respondent-level mean pain score was calculated across all scenarios as a descriptive summary of general rating tendency rather than a validated unidimensional scale. Internal consistency was assessed using Cronbach’s alpha (α).

Within each survey, ordinal outcomes were compared by sex using Mann–Whitney U tests and across years-in-practice categories using Kruskal–Wallis tests. Corresponding effect sizes were rank-biserial correlation (*r*) and epsilon-squared (ε^2^). Associations between attitudinal items and mean pain scores were assessed using Spearman’s rank correlation (ρ). The seven common attitudinal items were compared between surveys using Mann–Whitney U tests, with pointwise 95% bootstrap confidence intervals (CIs) for effect sizes. Four paired scenario contrasts per survey were assessed using Wilcoxon signed-rank tests. Between-survey comparisons also included six approximately analogous scenarios, with interpretation accounting for differences in species, wording, and clinical context.

Exploratory multivariable logistic regression models were fitted separately for each survey to examine factors associated with routinely discussing pain with producers. Q10 was coded as 1 for agree or strongly agree and 0 for neutral, disagree, or strongly disagree. Predictors were sex, years-in-practice category, emergency medical responder or volunteer firefighter status, and Q4–Q9 entered as 1-point Likert increments. Odds ratios (OR) and 95% CIs are reported in Table 1.

Descriptive distributions were emphasized, and all inferential analyses were considered exploratory. Benjamini–Hochberg false discovery rate (FDR) adjustment was applied separately by survey and analysis type. Sex and experience comparisons of attitudes each formed a seven-test family (Q4–Q10). For pain ratings, sex and experience comparisons were adjusted separately across all scenarios and the respondent-level mean score, yielding 11 outcomes for cattle and 10 for pigs. Attitude mean score correlations and paired scenario contrasts formed separate families of seven and four tests, respectively. Between-survey comparisons used separate families of seven common attitudinal items and six analogous scenarios. All tests were two-sided.

**Figure 1:**
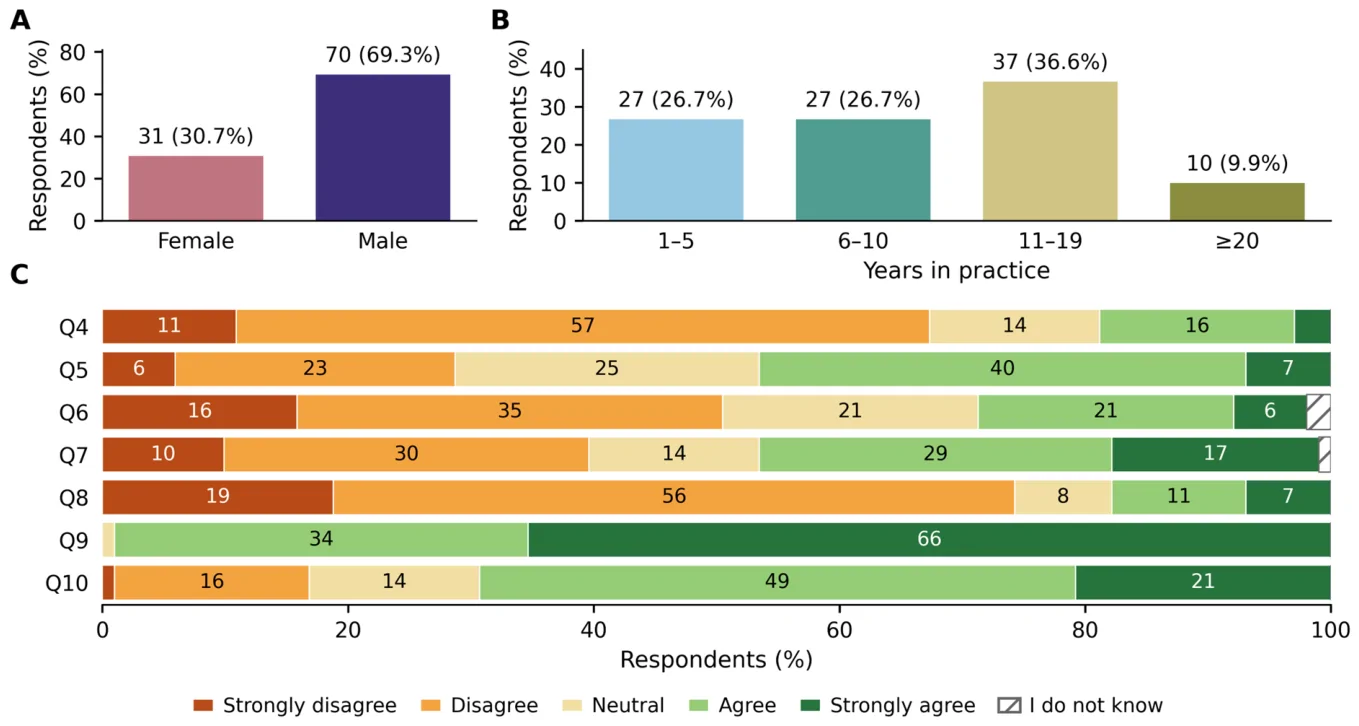
Cattle-focused survey: (**A**) sex, (**B**) years in practice, and (**C**) complete distributions of the seven common attitudinal items (*n* = 101). Demographic labels show counts and percentages. Stacked-bar segments represent the five ordered response categories and “I do not know.” Orange shades indicate disagreement, cream indicates a neutral response, and green shades indicate agreement. Hatched white segments indicate “I do not know.” Visible segment labels show counts of at least six. All percentages use the full denominator of 101. Q4–Q10 correspond to pain recognition, self-perceived knowledge, relative sensitivity to humans, analgesic availability, cost, recovery benefit, and producer communication, respectively. Exact wording and all category counts are provided in Supplementary File S1.

**Figure 2:**
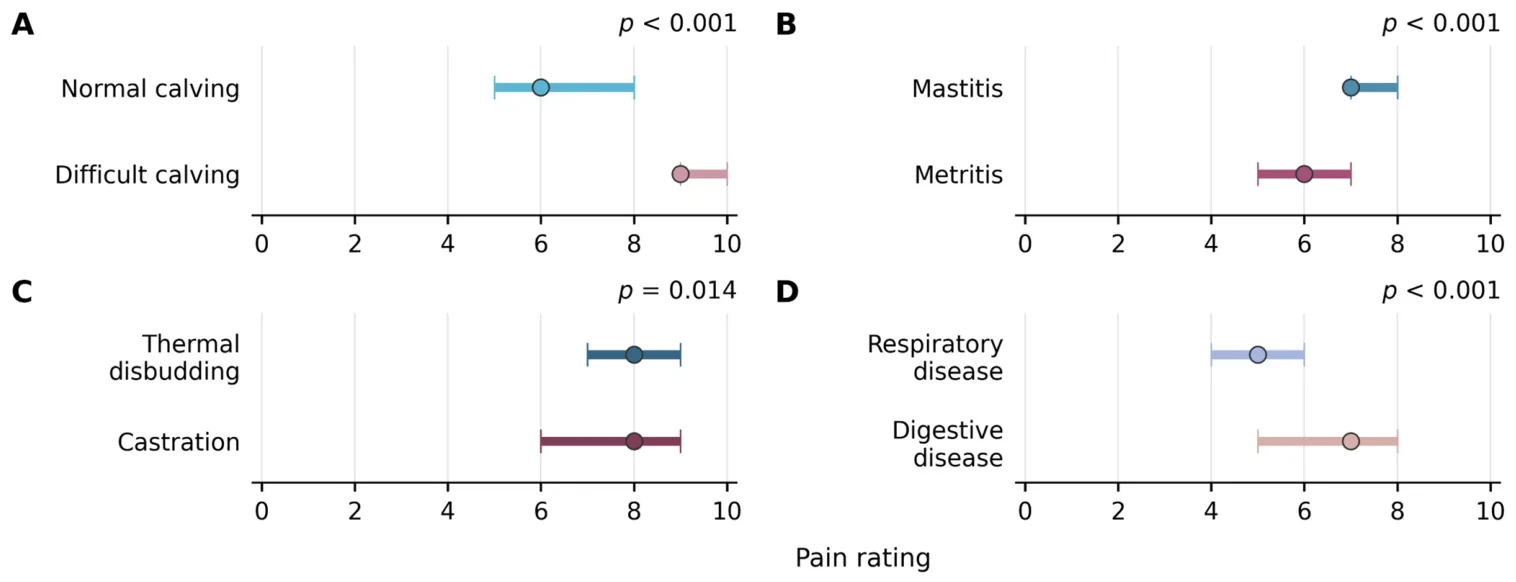
Cattle-focused survey: median and interquartile range of pain ratings for four exploratory paired contrasts (*n* = 101 per scenario). Points indicate medians and horizontal bars indicate IQR. Panels compare (**A**) normal and difficult calving, (**B**) mastitis and metritis, (**C**) thermal disbudding and castration, and (**D**) respiratory and digestive disease. Ratings use an integer NRS from 0 to 10.

**Figure 3:**
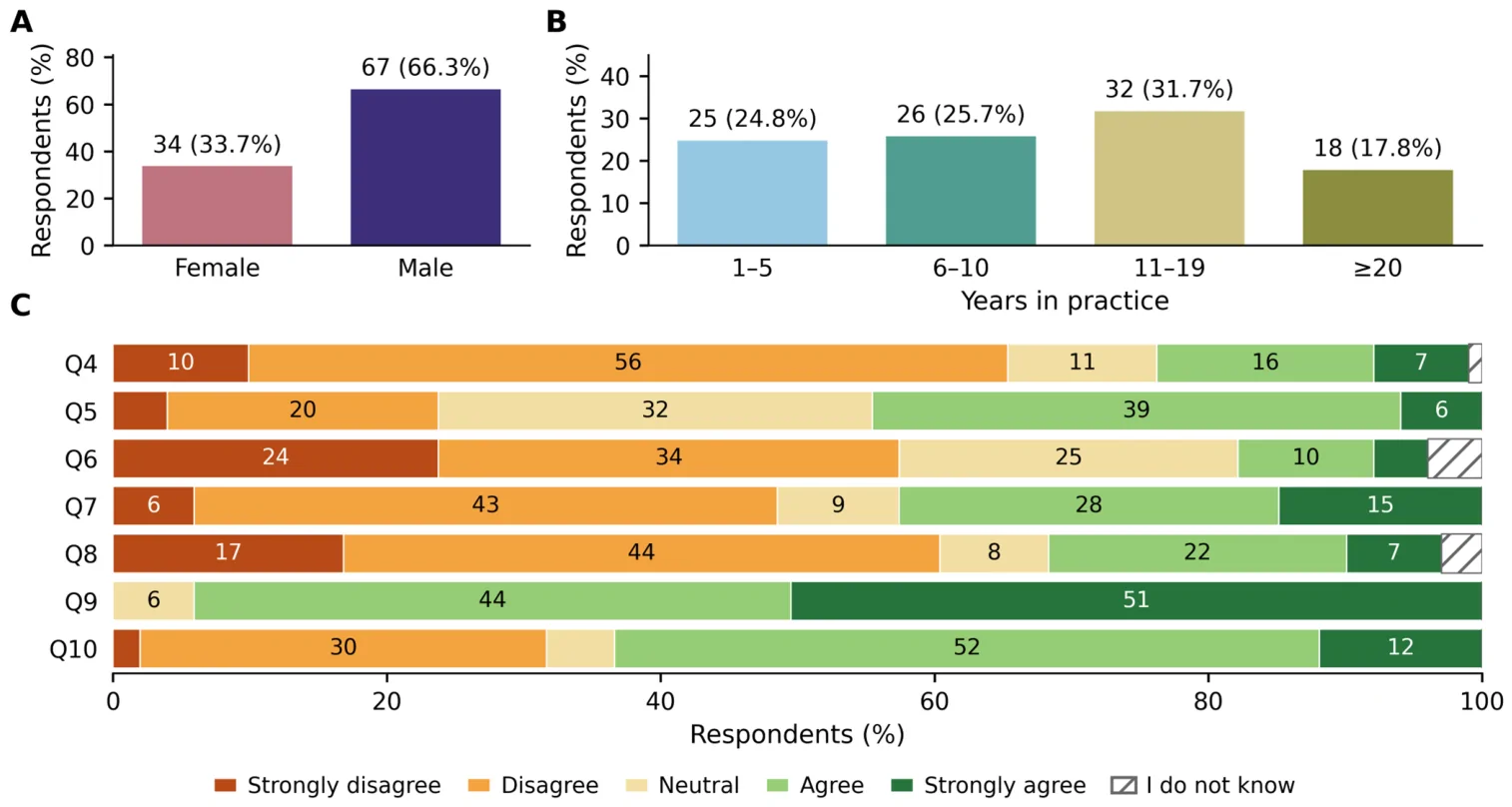
Pig-focused survey: (**A**) sex, (**B**) years in practice, and (**C**) complete distributions of the seven common attitudinal items (*n* = 101). Demographic labels show counts and percentages. Stacked-bar segments retain all five ordered response categories and “I do not know.” Orange shades indicate disagreement, cream indicates a neutral response, and green shades indicate agreement. Hatched white segments indicate “I do not know.” Visible segment labels show counts of at least six. Percentages use 101 as the denominator. Q4–Q10 correspond to pain recognition, self-perceived knowledge, relative sensitivity to humans, analgesic availability, cost, recovery benefit, and producer communication, respectively. Exact wording and all category counts are provided in Supplementary File S1.

**Figure 4:**
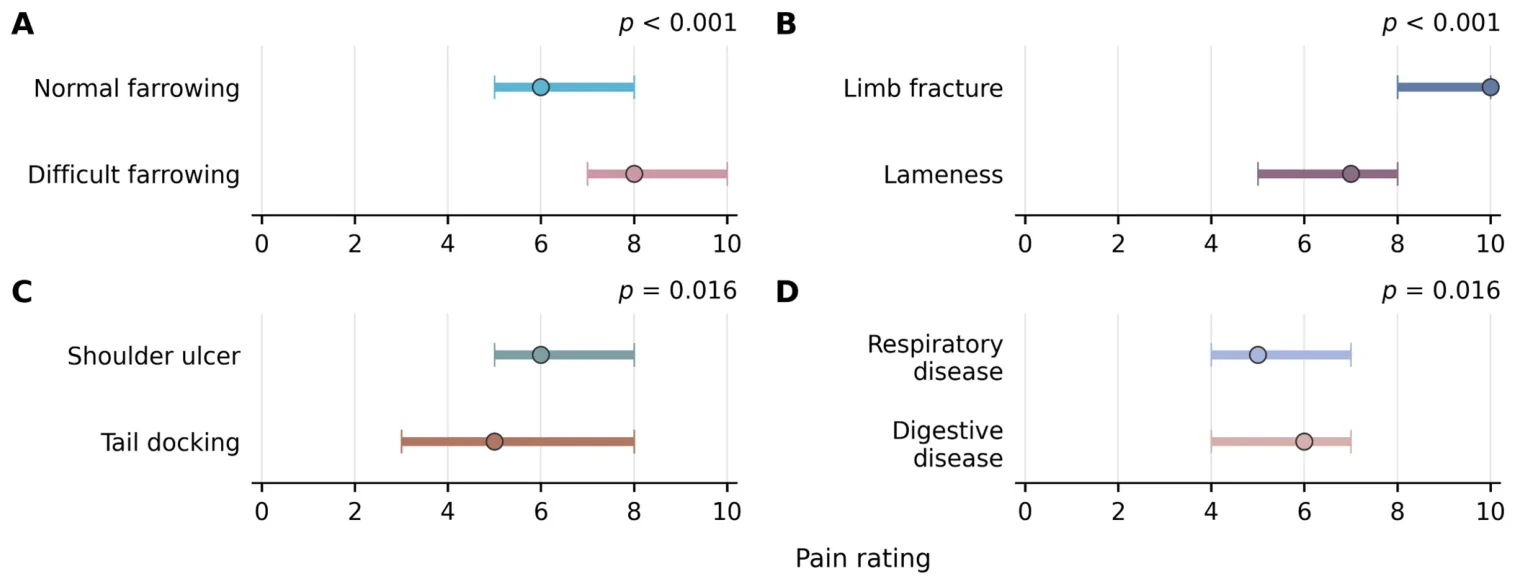
Pig-focused survey: median and interquartile range of pain ratings for four exploratory paired contrasts (n = 101 per scenario). Points indicate medians and horizontal bars indicate IQRs. Panels compare (**A**) normal and difficult farrowing, (**B**) limb fracture and lameness with minimal weight-bearing, (**C**) open shoulder ulcer and tail docking, and (**D**) respiratory and digestive disease. Ratings use an integer NRS from 0 to 10.

Figures were generated in Python 3.14 (Figure 1, Figure 2, Figure 3 and Figure 4) using Matplotlib 3.10 and NumPy 2.5. Figure 1 and Figure 3 present demographic counts and percentages and complete attitudinal response distributions, including “I do not know.” Percentages were calculated using all 101 respondents per survey. Figure 2 and Figure 4 present median pain ratings with IQR and FDR-adjusted *p* values for the paired scenario comparisons.

## 3. Results

### 3.1. Cattle-Focused Survey

#### 3.1.1. Sample Characteristics

A total of 101 cattle-focused responses were analyzed (data collection period: 30 March 2025–30 January 2026). Respondents were 70/101 male (69.3%) and 31/101 female (30.7%). Years in practice were most commonly 11–19 years (37/101; 36.6%), followed by 1–5 years (27/101; 26.7%), 6–10 years (27/101; 26.7%), and ≥20 years (10/101; 9.9%). Emergency medical responder/volunteer firefighter status was reported by 5/101 respondents (5%). Sex and years-in-practice distributions are shown in Figure 1A,B.

#### 3.1.2. Attitudes and Beliefs Regarding Pain Recognition and Pain Management

For the statement “It is difficult to recognize pain in cattle,” 68/101 respondents (67.3%) disagreed, 14/101 (13.9%) were neutral, and 19/101 (18.8%) agreed (median Likert score 2; IQR 2–3). For self-reported knowledge (“I feel that I know enough about pain and its treatment in cattle”), 47/101 (46.5%) agreed, 25/101 (24.8%) were neutral, and 29/101 (28.7%) disagreed (median 3; IQR 2–4). For the statement “Cattle are not as sensitive to pain as humans,” 51/101 (50.5%) disagreed, 21/101 (20.8%) were neutral, 27/101 (26.7%) agreed, and 2/101 (2.0%) selected “I do not know” (median 2; IQR 2–4; *n* = 99).

Perceived constraints on analgesic use were heterogeneous. For the statement “There are not enough analgesic drugs that can be used in cattle with painful conditions,” 46/101 (45.5%) agreed, 14/101 (13.9%) were neutral, 40/101 (39.6%) disagreed, and 1/101 (1.0%) selected “I do not know” (median 3; IQR 2–4; *n* = 100 scored). For “Analgesic drugs are too expensive for cattle producers to use routinely,” 75/101 (74.3%) disagreed, 8/101 (7.9%) were neutral, and 18/101 (17.8%) agreed (median 2; IQR 2–3). Endorsement of the clinical value of pain mitigation was near universal: for “Cattle recover better when pain is miti-gated,” 100/101 (99.0%) agreed and 1/101 (1.0%) was neutral (median 5; IQR 4–5). Routine discussion of pain with producers was reported by 70/101 respondents (69.3%) (median 4; IQR 3–4).

#### 3.1.3. Sex and Experience Effects on Attitudinal Items

After FDR adjustment within the seven-item sex-comparison family, female respondents more strongly endorsed insufficient analgesic options (median 4.0 vs. 3.0; rank-biserial *r* = 0.409; FDR-adjusted *p* = 0.006). Female respondents also more strongly endorsed the recovery benefit of pain mitigation, although both groups had a median of 5.0 (*r* = 0.270; FDR-adjusted *p* = 0.032). The latter finding concerns the response distribution rather than a difference in medians. No experience comparison remained significant after FDR adjustment. Complete item distributions are shown in Figure 1C, and subgroup statistics are provided in Supplementary File S1.

#### 3.1.4. Pain-Intensity Scenario Ratings

Perceived pain intensity varied markedly by scenario. Median (IQR) NRS ratings were 6 (5–8) for normal/physiological calving, 9 (9–10) for difficult calving requiring intervention, 7 (7–8) for mastitis, 5 (5–7) for recumbency, 7 (6–8) for lameness with minimal weight-bearing, 6 (5–7) for metritis, 5 (4–6) for respiratory disease, 7 (5–8) for digestive disease, 8 (7–9) for thermal disbudding, and 8 (6–9) for castration. Selected scenario medians and IQRs are shown in Figure 2A–D.

Severe pain ratings (≥8/10) were most frequent for difficult calving requiring intervention (88/101; 87.1%), including 10/10 ratings by 42/101 respondents (41.6%). Thermal disbudding was rated ≥8/10 by 69/101 respondents (68.3%), and castration by 60/101 (59.4%). The respondent-level mean pain score across all 10 scenarios had a median (IQR) of 6.9 (6.1–7.5) and a mean (SD) of 6.8 (1.0). Internal consistency across the 10 scenario ratings was α = 0.747.

Exploratory within-respondent comparisons of four conceptually linked scenario pairs (Figure 2A–D) showed higher ratings for difficult than normal calving (FDR-adjusted *p* < 0.001), mastitis than metritis (FDR-adjusted *p* < 0.001), disbudding than castration despite identical medians (FDR-adjusted *p* = 0.014), and digestive than respiratory disease (FDR-adjusted *p* < 0.001).

No sex- or years-in-practice differences in scenario pain ratings or in respondent-level mean pain scores remained statistically significant after FDR adjustment across the family of pain scenarios.

#### 3.1.5. Associations Between Attitudes and Respondent-Level Mean Pain Score

No Spearman correlation between attitudinal items (Q4–Q10) and the respondent-level mean pain score remained significant after FDR adjustment in the cattle-focused dataset.

#### 3.1.6. Exploratory Logistic Regression: Routine Discussion of Pain with Producers

In the cattle-focused dataset, the exploratory multivariable logistic model included 98 complete cases and was not significant by likelihood-ratio testing (*p* = 0.937; McFadden pseudo-R^2^ = 0.041). None of the predictor coefficients was statistically significant. All predictor ORs and 95% CIs are reported in Table 1.

### 3.2. Pig-Focused Survey

#### 3.2.1. Sample Characteristics

A total of 101 pig-focused responses were analyzed (data collection period: 27 March 2025–28 February 2026). Respondents were 67/101 male (66.3%) and 34/101 female (33.7%). Years in practice were 11–19 years for 32/101 respondents (31.7%), 6–10 years for 26/101 (25.7%), 1–5 years for 25/101 (24.8%), and ≥20 years for 18/101 (17.8%). Emergency medical responder/volunteer firefighter status was reported by 4/101 (4.0%). Sex and years-in-practice distributions are shown in Figure 3A,B.

#### 3.2.2. Attitudes and Beliefs Regarding Pain Recognition and Pain Management

For “It is difficult to recognize pain in pigs,” 66/101 respondents (65.3%) disagreed, 11/101 (10.9%) were neutral, 23/101 (22.8%) agreed, and 1/101 (1.0%) selected “I do not know” (median 2; IQR 2–3; *n* = 100 scored). For self-reported knowledge (“I feel that I know enough about pain and its treatment in pigs”), 45/101 (44.6%) agreed, 32/101 (31.7%) were neutral, and 24/101 (23.8%) disagreed (median 3; IQR 3–4). For “Pigs are not as sensitive to pain as humans,” 58/101 (57.4%) disagreed, 25/101 (24.8%) were neutral, 14/101 (13.9%) agreed, and 4/101 (4.0%) selected “I do not know” (median 2; IQR 2–3; *n* = 97 scored).

For “There are not enough analgesic drugs that can be used in pigs with painful conditions,” 43/101 (42.6%) agreed, 9/101 (8.9%) were neutral, and 49/101 (48.5%) disagreed (median 3; IQR 2–4). For “Analgesic drugs are too expensive for pig producers to use routinely,” 61/101 (60.4%) disagreed, 8/101 (7.9%) were neutral, 29/101 (28.7%) agreed, and 3/101 (3.0%) selected “I do not know” (median 2; IQR 2–4; *n* = 98 scored). For “Pigs recover better when pain is mitigated,” 95/101 (94.1%) agreed and 6/101 (5.9%) were neutral (median 5; IQR 4–5). Routine discussion of pain with producers was reported by 64/101 respondents (63.4%) (median 4; IQR 2–4).

#### 3.2.3. Sex and Experience Effects on Attitudinal Items

After FDR adjustment within the seven-item sex-comparison family, male respondents more strongly endorsed the statement “Pigs are not as sensitive to pain as humans” than female respondents (median 2.5 vs. 2.0; rank-biserial *r* = 0.341; FDR-adjusted *p* = 0.031). Complete item distributions are shown in Figure 3C. Subgroup statistics are provided in Supplementary File S1.

The overall distribution of perceived insufficiency of analgesic options differed across experience categories (Kruskal–Wallis H = 13.210; FDR-adjusted *p* = 0.029; ε^2^ = 0.105). The 11–19-year category had the lowest descriptive median (2.0; IQR 2.0–3.25), the other medians ranged from 3.0 to 4.0.

#### 3.2.4. Pain-Intensity Scenario Ratings

Median (IQR) NRS ratings were 6 (5–8) for normal/physiological farrowing, 8 (7–10) for difficult farrowing requiring internal examination, 8 (7–9) for infectious mastitis, 10 (8–10) for limb fracture, 7 (5–8) for lameness with minimal weight-bearing, 6 (5–8) for an open shoulder ulcer, 5 (4–7) for respiratory disease, 6 (4–7) for digestive disease, and 5 (3–8) for tail docking. Selected scenario medians and IQRs are shown in Figure 4A–D.

Limb fracture was most frequently rated as severe pain (≥8/10) (85/101; 84.2%), and 51/101 respondents (50.5%) rated fracture pain as 10/10. Tail-docking ratings varied substantially (IQR 3–8), with 27/101 respondents (26.7%) assigning ≥8/10 and 10/101 (9.9%) assigning 10/10. The respondent-level mean pain score across all 9 scenarios had a median (IQR) of 6.8 (5.9–7.7) and a mean (SD) of 6.7 (1.3). Internal consistency across the 9 scenario ratings was α = 0.829.

Exploratory within-respondent comparisons (Figure 4A–D) showed higher ratings for difficult than normal farrowing (medians 8 and 6; FDR-adjusted *p* < 0.001), limb fracture than lameness (10 and 7; FDR-adjusted *p* < 0.001), shoulder ulcer than tail docking (6 and 5; FDR-adjusted *p* = 0.016), and digestive than respiratory disease (6 and 5; FDR-adjusted *p* = 0.016).

#### 3.2.5. Associations Between Attitudes and Respondent-Level Mean Pain Score

After FDR adjustment across seven correlations, agreement that pigs are less sensitive to pain than humans was modestly associated with lower respondent-level mean pain ratings (Spearman ρ = −0.298; FDR-adjusted *p* = 0.021; *n* = 97). No other attitudinal item was significantly correlated with the mean pain score after adjustment.

#### 3.2.6. Exploratory Logistic Regression: Routine Discussion of Pain with Producers

In the pig-focused dataset, the exploratory multivariable logistic model included 93 complete cases and was significant by likelihood-ratio testing (*p* = 0.004; McFadden pseudo R^2^ = 0.234; in-sample AUC = 0.805). Affirmative endorsement of routine pain discussion was associated with higher self-reported knowledge (OR 2.11, 95% CI 1.13–3.93; *p* = 0.020) and stronger perceived insufficiency of analgesic options (OR 2.24, 95% CI 1.15–4.37; *p* = 0.018). The wide intervals for several other coefficients and sparse categories indicate limited precision. All predictor ORs and 95% CIs are reported in Table 1.

### 3.3. Exploratory Between-Survey Comparisons

In the seven common-item comparisons added during revision, no between-survey difference remained significant after FDR adjustment (Table 2). Rank-biserial effects ranged from −0.110 to 0.164.

In exploratory comparisons of six approximately analogous scenarios, difficult parturition was rated higher in the cattle-focused survey than in the pig-focused survey (median 9 vs. 8; FDR-adjusted *p* = 0.009), and digestive disease was rated higher in the cattle-focused survey (median 7 vs. 6; FDR-adjusted *p* = 0.024). No other analogous scenario differed after FDR adjustment.

## 4. Discussion

To our knowledge, this study is the first within-country comparison of pain perceptions and views on pain management among cattle- and pig-focused veterinarians in Poland. Veterinarians in both practice areas strongly endorsed the recovery benefits of pain relief and reported broadly similar patterns of responses to general pain-related statements. Variation was evident in ratings of the painfulness of selected procedures and clinical conditions, with additional associations linking specific pain-related attitudes to respondent characteristics and reported communication with producers.

Responses concerning general pain perception overlapped across the samples. Approximately two-thirds of respondents in each survey disagreed that pain was difficult to recognize, indicating self-reported confidence rather than verified assessment accuracy. Differences from an earlier pig survey [14] could reflect education, but this cross-sectional comparison cannot distinguish educational change from country, questionnaire wording, practice setting, recruitment, or respondent composition. Educational aspects of welfare and pain assessment have been discussed previously [21,22]. Pain assessment remains dependent on the species, condition, observer, and assessment method [1].

Approximately 45% of respondents endorsed sufficient knowledge of pain and its treatment; the remaining responses were neutral or indicated disagreement. Similar self-assessments have been reported previously [14,23]. This questionnaire measured perceived knowledge, not objective competence, and cannot demonstrate a knowledge deficit. These responses may inform assessment of perceived educational needs, but do not establish which educational intervention would be effective. Multiple clinical, behavioral, and physiological indicators contribute to pain assessment [8] and empathy may also influence pain ratings [15].

Most veterinarians disagreed that farm animals were less sensitive to pain than humans, although responses to this statement were more heterogeneous than responses to other pain-related statements. This finding is consistent with contemporary animal-welfare concepts that recognize animals as sentient beings capable of experiencing pain and suffering. Beliefs regarding animal sentience are closely related to attitudes toward animal welfare [24,25]. Direct comparisons of pain intensity among species remain inherently limited because pain is a subjective experience that cannot be measured directly [8].

Most respondents in both groups did not consider analgesics too expensive. This result is consistent with a survey of pig veterinarians in which 29% considered analgesics too expensive for routine use, whereas almost half disagreed [14]. Earlier studies of cattle practitioners identified greater concern about cost [26], but a subsequent survey indicated that perceived cost barriers had decreased and willingness to pay for analgesia had increased over time [16]. Other work has also suggested that cost is not among the most influential determinants of veterinarians’ decisions to administer nonsteroidal anti-inflammatory drugs, particularly among clinicians who already use analgesia routinely [27]. In the context of these earlier studies, the present findings may reflect a view of analgesia as an integral component of clinical care rather than an optional addition determined primarily by cost.

Economic considerations represent only one of several factors affecting analgesic use in farm animals. Other relevant factors include drug availability, authorization restrictions, veterinary knowledge, and organizational and legal conditions specific to food-producing animals [3]. Most pig veterinarians in the present study perceived the available analgesic options as sufficient, consistent with earlier observations [14]. Responses among cattle veterinarians were more heterogeneous. Perceived treatment options may depend not only on the number of authorized products but also on how veterinary services are organized. Although bovine and porcine practice share the principles of herd health management, the two fields differ in work organization, the scope of clinical decisions, and the nature of collaboration with producers [20]. The present survey assessed perceptions of cost and availability and did not measure product prices, the national availability of specific authorized products, producer willingness to pay, or prescribing behavior. Reported communication with producers likewise reflects self-reported discussions rather than directly observed clinical practice.

Regardless of perceived treatment limitations, veterinarians in both practice areas were nearly unanimous that effective analgesia supports animal recovery. Similar levels of agreement have been reported among pig and cattle veterinarians [14,16], and analgesia has been regarded as beneficial to both animals and producers [28]. Most respondents also reported routinely discussing pain with producers. Comparable communication rates were reported in an earlier survey of pig veterinarians [14]. Veterinarian–producer communication is a key component of effective herd health management because it influences implementation of recommendations, collaborative decision-making, and herd-care outcomes [29,30].

Veterinarians clearly differentiated the painfulness of individual procedures and clinical conditions. In both surveys, the highest ratings were assigned to severe injury, periparturient complications, and invasive procedures, whereas respiratory disease received the lowest ratings. This hierarchy resembles those reported in other surveys, in which severe injuries and invasive procedures received the highest scores, while physiological processes and less overtly painful disorders received lower scores [2,14,16].

Variation in ratings may reflect clinical experience, exposure frequency, production context, and interpretation of questionnaire wording, in addition to perceived nociceptive severity. Broad labels such as “digestive disease” and “respiratory disease” may lead respondents to consider different diagnoses, disease stages, or levels of severity. The level of detail provided in the questionnaire may also influence which clinical cases respondents recall from their practice and use as reference points. Ratings should therefore be interpreted as assessments of the scenarios as understood by the respondents. The paired scenario contrasts were exploratory and should be considered in this context.

Normal parturition was not considered a minimally painful condition. Median ratings of 6/10 for both calving and farrowing indicate that respondents recognized appreciable pain during physiological parturition. Uterine contractions, cervical dilation, and stretching of birth-canal tissues contribute to pain despite endogenous hypoalgesic mechanisms [31,32]. Respondents consistently rated obstetric intervention as more painful than uncomplicated parturition, consistent with previous surveys [14,16]. Prolonged labor, tissue trauma, and obstetric manipulation may help explain the higher ratings assigned to dystocia [31].

Recumbency in cattle received a moderate pain rating. Interpretation is difficult because recumbency is a clinical sign rather than a single disease and can result from disorders with diverse etiologies and pain severity. Respondents may therefore have considered different causes of recumbency. Analgesic treatment is considered an important component of supportive care for nonambulatory cows [33,34].

Cattle veterinarians rated mastitis as more painful than metritis. The difference may reflect the more overt clinical expression of pain during acute mammary inflammation, including discomfort during palpation and milking [35,36,37]. The lower rating assigned to metritis does not imply that the disease was considered only mildly painful. Metritis is associated primarily with visceral pain, whose clinical expression may be less readily apparent during routine examination than pain associated with mastitis [38].

Among pig veterinarians, infectious mastitis was one of the more highly rated clinical conditions, consistent with previous findings among veterinarians and pig producers [14]. Pain associated with mammary inflammation may restrict nursing and alter maternal behavior, although a recent review concluded that the topic remains insufficiently characterized [39].

Limb fracture received the highest pain ratings among all clinical conditions evaluated in pigs. More than half of respondents assigned the maximum rating of 10/10, and more than 84% assigned a rating of at least 8/10. Fractures were also among the most painful injuries in a previous survey [14]. Fracture pain is associated with tissue injury, inflammation, and instability of bone fragments [40].

Pain ratings for shoulder ulcers in pigs were similar to those in an earlier survey, in which the condition also received a median rating of 6 [14]. These lesions can involve deeper tissues and cause persistent pain associated with inflammation and neural injury [41]. The ratings indicate recognition of pain associated with this chronic condition, which has received relatively little attention in studies of pain perception [39].

Lameness received high pain ratings from both cattle and pig veterinarians (median, 7/10). Similar ratings have been reported in other surveys, in which lameness consistently ranked among the most painful conditions in farm animals [14,16]. The importance of lameness for cattle welfare is reflected in research on behavior and productivity and the use of validated locomotion-scoring systems. Contemporary guidance recognizes pain as a fundamental clinical component of lameness and appropriate analgesia as an integral part of treatment [42].

Respiratory disease received an identical median pain rating of 5/10 in both surveys, whereas digestive disease received slightly higher ratings of 6–7/10. Previous surveys have likewise shown that respiratory disease is perceived as painful in cattle and pigs, although ratings are lower than those assigned to trauma or severe inflammation [14,16]. Digestive disease received ratings approaching those for mastitis and lameness, suggesting that respondents also considered pain an important component of internal medical disorders.

Routine husbandry and surgical procedures involve predictable tissue injury. Thermal disbudding and cattle castration received the highest ratings among the evaluated procedures (median, 8/10 for both), whereas piglet tail docking received lower and substantially more variable ratings. Experimental studies have documented pain following disbudding and castration and shown that local anesthesia combined with a nonsteroidal anti-inflammatory drug reduces the pain response [43,44,45]. The high ratings assigned to these procedures are therefore consistent with current evidence supporting peri-procedural pain management in calves.

Piglet tail docking received a lower pain rating than the procedures evaluated in cattle (median, 5/10). Tail docking is commonly performed in piglets during the first days of life [10,46]. Although painful procedures performed early in life were historically assumed to cause less pain because of nervous system immaturity, current evidence does not support this assumption [47,48]. Peripheral nociceptive pathways are functional in neonates, while later maturation of inhibitory mechanisms may contribute to increased sensitivity [47,48]. Experimental studies have shown that tail docking elicits immediate vocal, behavioral, and physiological responses consistent with acute pain and can result in traumatic neuroma formation within the tail stump, with possible implications for persistent sensitivity [46,49,50].

Pain assessment in animals is not based solely on knowledge of the pathophysiology of individual conditions. It also includes cognitive and affective components that can be influenced by veterinarian characteristics, including empathy [15]. In the present study, selected attitude differences remained significant after false discovery rate adjustment. Female cattle veterinarians more strongly endorsed insufficient analgesic options, whereas male pig veterinarians more strongly endorsed the belief that pigs are less sensitive to pain than humans. The stronger endorsement of the value of pain mitigation for recovery among female cattle veterinarians was a nominal finding that did not remain significant after adjustment. Among pig veterinarians, stronger endorsement of lower pain sensitivity in pigs was modestly associated with lower respondent-level mean pain ratings (ρ = −0.298). The observed pattern is consistent with the multidimensional nature of pain assessment described previously [15]. No sex-related differences in scenario-specific pain ratings remained significant after multiplicity adjustment.

Comparable surveys have yielded mixed findings. A UK survey of farmers and veterinarians found higher overall pain scores among younger than older respondents [14], while UK cattle practitioners who graduated before 1990 assigned lower pain scores and were less likely to use nonsteroidal anti-inflammatory drugs than more recent graduates [16]. More recent veterinary graduates also assigned higher disbudding pain scores in a New Zealand survey [17]. Conversely, professional experience was not associated with mean cattle pain scores in a Finnish survey [15], and age-related differences in a U.S. cattle survey were limited to selected procedures and showed no consistent pattern [18]. Taken together, career stage may be associated with perceptions of particular procedures or treatment decisions but does not consistently predict pain ratings across settings.

The purposive non-probability sampling and absence of a sampling frame prevented calculation of a response rate or population coverage. No reliable denominator for veterinarians practicing predominantly in cattle or pig medicine was available. The samples of 101 responses per survey cannot be assumed to represent the corresponding Polish professional populations. Self-selection may have favored veterinarians interested in pain or welfare, and social-desirability bias is possible. Subgroup sizes were small. The questionnaires did not quantify practice composition, and anonymity prevented exclusion of participation in both surveys by the same individual, potentially violating the independence assumption. Responses were collected over several months.

The six-person pilot established feasibility only, not formal content validity or measurement validity. The survey measured self-reported beliefs and communication rather than observed prescribing or objective knowledge. “I do not know” may reflect meaningful uncertainty. Complete case exclusion could introduce bias if these responses were not missing at random. Cronbach’s alpha does not establish unidimensionality of heterogeneous clinical scenarios, and the mean score is a pragmatic summary of rating tendency. Dichotomizing loses ordinal information, and linear Likert predictors impose an unverified log-odds assumption. The regression models are parameter-rich relative to the samples and include sparse categories, particularly emergency responder or firefighter status, creating a risk of overfitting and imprecise estimates. Approximate cross-species scenario comparisons may reflect wording or clinical-context differences. Pointwise bootstrap CIs for the new common-item comparisons do not establish equivalence or remedy sampling bias. The cross-sectional design precludes causal interpretation.

## 5. Conclusions

Descriptive response patterns among participating cattle and pig veterinarians in Poland suggested broadly similar attitudes toward pain recognition and analgesia, including strong agreement that pain mitigation supports recovery and frequent self-reported discussion of pain with producers. Pain ratings nevertheless varied substantially across conditions and procedures. Difficult calving, thermal disbudding, and castration were rated as particularly painful in cattle, whereas limb fracture received the highest ratings in pigs and tail-docking ratings were highly variable. Selected attitudes were associated with sex and professional experience. Uncertainty in self-perceived knowledge and variation in pain assessment and perceived treatment options identify areas that may warrant attention in continuing education, practical species-specific analgesic guidance, and veterinarian–producer communication.

## Figures and Tables

**Table 1:** Exploratory logistic-regression predictor effects for affirmative endorsement of routine pain discussion with producers.

Predictor	Cattle: OR (95% CI); *p*	Pigs: OR (95% CI); *p*
Years 6–10 vs. 1–5	0.99 (0.27–3.53); 0.982	0.43 (0.09–2.11); 0.299
Years 11–19 vs. 1–5	0.76 (0.21–2.75); 0.676	0.34 (0.07–1.56); 0.163
Years ≥20 vs. 1–5	0.42 (0.06–2.90); 0.382	0.34 (0.05–2.21); 0.259
Female vs. male	0.47 (0.14–1.51); 0.205	3.20 (0.86–11.86); 0.082
Emergency responder/firefighter	0.81 (0.11–6.08); 0.837	0.06 (0.00–1.10); 0.058
Q4 Difficult to recognize pain	0.90 (0.56–1.46); 0.679	1.16 (0.66–2.03); 0.608
Q5 Sufficient knowledge	1.25 (0.78–1.99); 0.357	2.11 (1.13–3.93); 0.020
Q6 Less sensitive than humans	0.95 (0.62–1.48); 0.836	0.82 (0.48–1.42); 0.478
Q7 Insufficient analgesic options	1.12 (0.71–1.75); 0.630	2.24 (1.15–4.37); 0.018
Q8 Analgesics too expensive	0.83 (0.53–1.28); 0.397	0.59 (0.31–1.10); 0.095
Q9 Recovery benefit	1.95 (0.74–5.13); 0.174	2.37 (0.96–5.85); 0.061

Reference categories: male, 1–5 years in practice, and no emergency responder/firefighter status. Cattle: *n* = 98, likelihood-ratio *p* = 0.937, McFadden R^2^ = 0.041. Pigs: *n* = 93, likelihood-ratio *p* = 0.004, McFadden R^2^ = 0.234, in-sample AUC = 0.805.

**Table 2:** Exploratory comparisons of the seven common attitudinal items, added during revision.

Item	Cattle *n*; Median (IQR)	Pigs *n*; Median (IQR)	r (95% CI)	FDR-Adjusted *p*
Q4 Difficult to recognize pain	101; 2 (2–3)	100; 2 (2–3)	−0.035 (−0.180 to 0.109)	0.738
Q5 Sufficient self-reported knowledge	101; 3 (2–4)	101; 3 (3–4)	−0.012 (−0.165 to 0.140)	0.880
Q6 Less sensitive than humans	99; 2 (2–4)	97; 2 (2–3)	0.151 (−0.005 to 0.301)	0.138
Q7 Insufficient analgesic options	100; 3 (2–4)	101; 3 (2–4)	0.046 (−0.109 to 0.199)	0.738
Q8 Analgesics too expensive	101; 2 (2–3)	98; 2 (2–4)	−0.110 (−0.255 to 0.040)	0.264
Q9 Pain mitigation supports recovery	101; 5 (4–5)	101; 5 (4–5)	0.164 (0.028 to 0.299)	0.138
Q10 Routine pain discussion	101; 4 (3–4)	101; 4 (2–4)	0.145 (−0.001 to 0.288)	0.138

“I do not know” responses were excluded from numeric analyses. CIs are pointwise, unadjusted 95% percentile bootstrap intervals. Benjamini–Hochberg adjustment was applied to the seven two-sided Mann–Whitney tests.

## Data Availability

The data supporting the findings of this study are available from the corresponding author upon reasonable request. The questionnaires in the original Polish and in English translation, together with item-level statistical results, are provided in Supplementary File S1.

## Supplementary Materials

The following supporting information can be downloaded at: https://www.mdpi.com/article/10.3390/ani16203199/s1, File S1: Veterinary practitioner surveys on pain perception and pain management in cattle and pigs: Polish-language questionnaires with English translations and item-level statistical results.

## Abbreviations

The following abbreviations are used in this manuscript:

FDR	false discovery rate
IQR	interquartile range
NRS	numeric rating scale
SD	standard deviation
