# Supplementary material for: Veterinarians’ Perceptions of Farm Animal Pain and Views on Pain Management: Two Cross-Sectional Scenario-Based Surveys of Cattle and Pig Practitioners in Poland
Source: Animals (Basel). 2026 Oct 12;16(20):3199. doi: 10.3390/ani16203199 (PMC13700574; doi:10.3390/ani16203199)
Supplement: Supplementary file 1 [file animals-16-03199-s001.zip › animals-4548153-supplementary File S1.pdf]

Supplementary Materials

Veterinary practitioner surveys on pain perception and pain management in cattle and pigs: Polish-language questionnaires with English translations and item-level statistical results.

S1. Survey instruments

For attitudinal items (Q4–Q10), respondents selected one response on a 5-point Likert scale with an additional “I do not know” option. For statistical scoring, responses were coded from 1 (strongly disagree) to 5 (strongly agree); “I do not know” responses were treated as missing for analyses requiring numeric scoring.

For pain-intensity scenarios (cattle Q11–Q20; pigs Q11–Q19), respondents rated perceived pain intensity on an 11-point numeric rating scale (NRS) ranging from 0 (no pain) to 10 (most severe pain).

S1.1 Cattle-focused questionnaire

Q1. Płeć:

Sex

Response options:

- Male
- Female

Q2. Ile lat pracujesz jako praktyk w zawodzie lekarza weterynarii?

Years in veterinary practice

Response options:

- 1–5 years
- 6–10 years
- 11–19 years
- ≥20 years

Q3. Czy jesteś również Ratownikiem Medycznym lub członkiem Ochotniczej Straży Pożarnej?

Emergency medical responder or volunteer firefighter status

Response options:

- Yes

- No

Q4. Trudno rozpoznać ból u bydła.

It is difficult to recognize pain in cattle.

Response options:

- Strongly disagree
- Disagree
- Neither agree nor disagree
- Agree
- Strongly agree
- I do not know

Q5. Czuję, że wiem wystarczająco dużo o bólu i sposobach jego leczenia u bydła.

I feel that I know enough about pain and its treatment in cattle.

Response options:

- Strongly disagree
- Disagree
- Neither agree nor disagree
- Agree
- Strongly agree
- I do not know

Q6. Bydło nie jest tak wrażliwe na ból jak ludzie.

Cattle are not as sensitive to pain as humans.

Response options:

- Strongly disagree
- Disagree
- Neither agree nor disagree
- Agree

- Strongly agree
- I do not know

Q7. Nie ma wystarczającej liczby leków przeciwbólowych, które można stosować u bydła cierpiącego na bolesne schorzenia.

There are not enough analgesic drugs that can be used in cattle with painful conditions.

Response options:

- Strongly disagree
- Disagree
- Neither agree nor disagree
- Agree
- Strongly agree
- I do not know

Q8. Leki przeciwbólowe są zbyt drogie, aby hodowcy bydła mogli je regularnie stosować.

Analgesic drugs are too expensive for cattle producers to use routinely.

Response options:

- Strongly disagree
- Disagree
- Neither agree nor disagree
- Agree
- Strongly agree
- I do not know

Q9. Bydło lepiej wraca do zdrowia dzięki łagodzeniu bólu.

Cattle recover better when pain is mitigated.

Response options:

- Strongly disagree
- Disagree
- Neither agree nor disagree

- Agree
- Strongly agree
- I do not know

Q10. Regularnie omawiam ból i opcje łagodzenia bólu z hodowcami bydła.

I routinely discuss pain and pain-mitigation options with cattle producers.

Response options:

- Strongly disagree
- Disagree
- Neither agree nor disagree
- Agree
- Strongly agree
- I do not know

Q11. Według Twojej oceny podczas normalnego/fizjologicznego porodu w skali od 0 (brak bólu) do 10 (najsilniejszy ból) krowy odczuwają ból w stopniu równym:

In your opinion, during normal/physiological calving, cows experience pain rated as (0–10).

Response format: Integer 0–10 (0 = no pain; 10 = most severe pain).

Q12. Według Twojej oceny podczas trudnego porodu wymagającego interwencji w skali od 0 (brak bólu) do 10 (najsilniejszy ból) krowy odczuwają ból w stopniu równym:

In your opinion, during difficult calving requiring intervention, cows experience pain rated as (0–10).

Response format: Integer 0–10 (0 = no pain; 10 = most severe pain).

Q13. Według Twojej oceny podczas zapalenia wymienia w skali od 0 (brak bólu) do 10 (najsilniejszy ból) krowy odczuwają ból w stopniu równym:

In your opinion, during mastitis, cows experience pain rated as (0–10).

Response format: Integer 0–10 (0 = no pain; 10 = most severe pain).

Q14. Według Twojej oceny podczas zalegania w skali od 0 (brak bólu) do 10 (najsilniejszy ból) krowy odczuwają ból w stopniu równym:

In your opinion, during recumbency, cows experience pain rated as (0–10).

Response format: Integer 0–10 (0 = no pain; 10 = most severe pain).

Q15. Według Twojej oceny podczas kulawizny (minimalne obciążenie) w skali od 0 (brak bólu) do 10 (najsilniejszy ból) bydło odczuwa ból w stopniu równym:

In your opinion, during lameness (minimal weight-bearing), cattle experience pain rated as (0–10).

Response format: Integer 0–10 (0 = no pain; 10 = most severe pain).

Q16. Według Twojej oceny podczas zapalenia macicy w skali od 0 (brak bólu) do 10 (najsilniejszy ból) krowy odczuwają ból w stopniu równym:

In your opinion, during metritis, cows experience pain rated as (0–10).

Response format: Integer 0–10 (0 = no pain; 10 = most severe pain).

Q17. Według Twojej oceny podczas chorób układu oddechowego w skali od 0 (brak bólu) do 10 (najsilniejszy ból) bydło odczuwa ból w stopniu równym:

In your opinion, during respiratory disease, cattle experience pain rated as (0–10).

Response format: Integer 0–10 (0 = no pain; 10 = most severe pain).

Q18. Według Twojej oceny podczas chorób układu pokarmowego w skali od 0 (brak bólu) do 10 (najsilniejszy ból) bydło odczuwa ból w stopniu równym:

In your opinion, during digestive disease, cattle experience pain rated as (0–10).

Response format: Integer 0–10 (0 = no pain; 10 = most severe pain).

Q19. Według Twojej oceny podczas dekornizacji metodą termiczną w skali od 0 (brak bólu) do 10 (najsilniejszy ból) cielęta odczuwają ból w stopniu równym:

In your opinion, during thermal disbudding, calves experience pain rated as (0–10).

Response format: Integer 0–10 (0 = no pain; 10 = most severe pain).

Q20. Według Twojej oceny podczas kastracji w skali od 0 (brak bólu) do 10 (najsilniejszy ból) buhaje odczuwają ból w stopniu równym:

In your opinion, during castration, bulls experience pain rated as (0–10).

Response format: Integer 0–10 (0 = no pain; 10 = most severe pain).

S1.2 Pig-focused questionnaire

Q1. Płeć:

Sex

Response options:

- Male
- Female

Q2. Ile lat pracujesz jako praktyk w zawodzie lekarza weterynarii?

Years in veterinary practice

Response options:

- 1–5 years
- 6–10 years
- 11–19 years
- ≥20 years

Q3. Czy jesteś również Ratownikiem Medycznym lub członkiem Ochotniczej Straży Pożarnej?

Emergency medical responder or volunteer firefighter status

Response options:

- Yes
- No

Q4. Trudno rozpoznać ból u świni.

It is difficult to recognize pain in pigs.

Response options:

- Strongly disagree
- Disagree
- Neither agree nor disagree
- Agree
- Strongly agree

- I do not know

Q5. Czuję, że wiem wystarczająco dużo o bólu i sposobach jego leczenia u świń.

I feel that I know enough about pain and its treatment in pigs.

Response options:

- Strongly disagree
- Disagree
- Neither agree nor disagree
- Agree
- Strongly agree
- I do not know

Q6. Świnie nie są tak wrażliwe na ból jak ludzie.

Pigs are not as sensitive to pain as humans.

Response options:

- Strongly disagree
- Disagree
- Neither agree nor disagree
- Agree
- Strongly agree
- I do not know

Q7. Nie ma wystarczającej liczby leków przeciwbólowych, które można stosować u świń cierpiących na

bolesne schorzenia.

There are not enough analgesic drugs that can be used in pigs with painful conditions.

Response options:

- Strongly disagree
- Disagree
- Neither agree nor disagree

- Agree
- Strongly agree
- I do not know

Q8. Leki przeciwbólowe są zbyt drogie, aby hodowcy trzody chlewnej mogli je regularnie stosować.

Analgesic drugs are too expensive for pig producers to use routinely.

Response options:

- Strongly disagree
- Disagree
- Neither agree nor disagree
- Agree
- Strongly agree
- I do not know

Q9. Świnie lepiej wracają do zdrowia dzięki łagodzeniu bólu.

Pigs recover better when pain is mitigated.

Response options:

- Strongly disagree
- Disagree
- Neither agree nor disagree
- Agree
- Strongly agree
- I do not know

Q10. Regularnie omawiam ból i opcje łagodzenia bólu z hodowcami świń.

I routinely discuss pain and pain-mitigation options with pig producers.

Response options:

- Strongly disagree
- Disagree

- Neither agree nor disagree
- Agree
- Strongly agree
- I do not know

Q11. Według Twojej oceny podczas normalnego/fizjologicznego porodu w skali od 0 (brak bólu) do 10 (najsilniejszy ból) lochy odczuwają ból w stopniu równym:

In your opinion, during normal/physiological farrowing, sows experience pain rated as (0–10).

Response format: Integer 0–10 (0 = no pain; 10 = most severe pain).

Q12. Według Twojej oceny podczas trudnego porodu wymagającego badania wewnętrznego w skali od 0 (brak bólu) do 10 (najsilniejszy ból) lochy odczuwają ból w stopniu równym:

In your opinion, during difficult farrowing requiring internal examination, sows experience pain rated as (0–10).

Response format: Integer 0–10 (0 = no pain; 10 = most severe pain).

Q13. Według Twojej oceny podczas zapalenia sutka na tle zakaźnym w skali od 0 (brak bólu) do 10 (najsilniejszy ból) lochy odczuwają ból w stopniu równym:

In your opinion, during infectious mastitis, sows experience pain rated as (0–10).

Response format: Integer 0–10 (0 = no pain; 10 = most severe pain).

Q14. Według Twojej oceny podczas złamania kończyny w skali od 0 (brak bólu) do 10 (najsilniejszy ból) świnie odczuwają ból w stopniu równym:

In your opinion, during limb fracture, pigs experience pain rated as (0–10).

Response format: Integer 0–10 (0 = no pain; 10 = most severe pain).

Q15. Według Twojej oceny podczas kulawizny (minimalne obciążenie) w skali od 0 (brak bólu) do 10 (najsilniejszy ból) świnie odczuwają ból w stopniu równym:

In your opinion, during lameness (minimal weight-bearing), pigs experience pain rated as (0–10).

Response format: Integer 0–10 (0 = no pain; 10 = most severe pain).

Q16. Według Twojej oceny podczas owrzodzenia barku (rana otwarta) w skali od 0 (brak bólu) do 10 (najsilniejszy ból) świnie odczuwają ból w stopniu równym:

In your opinion, during an open shoulder ulcer, pigs experience pain rated as (0–10).

Response format: Integer 0–10 (0 = no pain; 10 = most severe pain).

Q17. Według Twojej oceny podczas chorób układu oddechowego w skali od 0 (brak bólu) do 10 (najsilniejszy ból) świnie odczuwają ból w stopniu równym:

In your opinion, during respiratory disease, pigs experience pain rated as (0–10).

Response format: Integer 0–10 (0 = no pain; 10 = most severe pain).

Q18. Według Twojej oceny podczas chorób układu pokarmowego w skali od 0 (brak bólu) do 10 (najsilniejszy ból) świnie odczuwają ból w stopniu równym:

In your opinion, during digestive disease, pigs experience pain rated as (0–10).

Response format: Integer 0–10 (0 = no pain; 10 = most severe pain).

Q19. Według Twojej oceny podczas przycinania ogonków w skali od 0 (brak bólu) do 10 (najsilniejszy ból) prosięta odczuwają ból w stopniu równym:

In your opinion, during tail docking, piglets experience pain rated as (0–10).

Response format: Integer 0–10 (0 = no pain; 10 = most severe pain).

S2. Item-level statistical results

All descriptive and inferential statistics below were computed directly from the raw survey exports. Percentages use the full survey denominator (n = 101 for each survey) unless otherwise stated. For Likert items, "I do not know" responses were reported descriptively but treated as missing for numeric scoring and inferential testing. For inferential testing, Benjamini–Hochberg false discovery rate (FDR) adjustment was applied within each family of related hypotheses (attitudinal items; pain-scenario ratings).

S2.1 Cattle survey

Demographics

Total responses analyzed: 101.

Sex: 70/101 male (69.3%), 31/101 female (30.7%).

Years in practice: 27/101 (26.7%) 1-5 years; 27/101 (26.7%) 6-10 years; 37/101 (36.6%) 11-19 years; 10/101 (9.9%) ≥20 years.

Emergency medical responder/volunteer firefighter: 5/101 (5.0%).

S2.1.1 Attitudinal items (Q4–Q10)

Q4. It is difficult to recognize pain in cattle.

Strongly disagree: 11/101 (10.9%); Disagree: 57/101 (56.4%); Neutral: 14/101 (13.9%); Agree: 16/101 (15.8%); Strongly agree: 3/101 (3.0%).

Likert score (1–5): median (IQR) 2 (2–3) (excluding "I do not know").

Sex comparison (female vs male; Mann–Whitney U): $U = 1028.5$; $p = 0.647328$; FDR $q = 0.755216$; rank-biserial $r = 0.0521$.

Years-in-practice comparison (Kruskal–Wallis): $H = 5.780$; $p = 0.122810$; FDR $q = 0.413851$; $\varepsilon^2 = 0.029$.

Q5. I feel that I know enough about pain and its treatment in cattle.

Strongly disagree: 6/101 (5.9%); Disagree: 23/101 (22.8%); Neutral: 25/101 (24.8%); Agree: 40/101 (39.6%); Strongly agree: 7/101 (6.9%).

Likert score (1–5): median (IQR) 3 (2–4) (excluding "I do not know").

Sex comparison (female vs male; Mann–Whitney U): $U = 941.5$; $p = 0.269815$; FDR $q = 0.472177$; rank-biserial $r = 0.1323$.

Years-in-practice comparison (Kruskal–Wallis): $H = 2.839$; $p = 0.417102$; FDR $q = 0.729928$; $\varepsilon^2 = -0.002$.

Q6. Cattle are not as sensitive to pain as humans.

Strongly disagree: 16/101 (15.8%); Disagree: 35/101 (34.7%); Neutral: 21/101 (20.8%); Agree: 21/101 (20.8%); Strongly agree: 6/101 (5.9%). "I do not know": 2/101 (2.0%).

Likert score (1–5): median (IQR) 2 (2–4) (excluding "I do not know").

Sex comparison (female vs male; Mann–Whitney U): $U = 820.5$; $p = 0.068667$; FDR $q = 0.160222$; rank-biserial $r = 0.2215$.

Years-in-practice comparison (Kruskal–Wallis): $H = 0.853$; $p = 0.836713$; FDR $q = 0.958632$; $\varepsilon^2 = -0.023$.

Q7. There are not enough analgesic drugs that can be used in cattle with painful conditions.

Strongly disagree: 10/101 (9.9%); Disagree: 30/101 (29.7%); Neutral: 14/101 (13.9%); Agree: 29/101 (28.7%); Strongly agree: 17/101 (16.8%). “I do not know”: 1/101 (1.0%).

Likert score (1–5): median (IQR) 3 (2–4) (excluding “I do not know”).

Sex comparison (female vs male; Mann–Whitney U): $U = 632.5$; $p = 0.000793$; FDR $q = 0.005549$; rank-biserial $r = 0.4086$.

Years-in-practice comparison (Kruskal–Wallis): $H = 7.383$; $p = 0.060644$; FDR $q = 0.413851$; $\varepsilon^2 = 0.046$.

Q8. Analgesic drugs are too expensive for cattle producers to use routinely.

Strongly disagree: 19/101 (18.8%); Disagree: 56/101 (55.4%); Neutral: 8/101 (7.9%); Agree: 11/101 (10.9%); Strongly agree: 7/101 (6.9%).

Likert score (1–5): median (IQR) 2 (2–3) (excluding “I do not know”).

Sex comparison (female vs male; Mann–Whitney U): $U = 1007.0$; $p = 0.528799$; FDR $q = 0.740319$; rank-biserial $r = 0.0719$.

Years-in-practice comparison (Kruskal–Wallis): $H = 2.212$; $p = 0.529491$; FDR $q = 0.741287$; $\varepsilon^2 = -0.008$.

Q9. Cattle recover better when pain is mitigated.

Strongly disagree: 0/101 (0.0%); Disagree: 0/101 (0.0%); Neutral: 1/101 (1.0%); Agree: 34/101 (33.7%); Strongly agree: 66/101 (65.3%).

Likert score (1–5): median (IQR) 5 (4–5) (excluding “I do not know”).

Sex comparison (female vs male; Mann–Whitney U): $U = 792.5$; $p = 0.009274$; FDR $q = 0.032459$; rank-biserial $r = 0.2696$.

Years-in-practice comparison (Kruskal–Wallis): $H = 4.925$; $p = 0.177365$; FDR $q = 0.413851$; $\varepsilon^2 = 0.020$.

Q10. I routinely discuss pain and pain-mitigation options with cattle producers.

Strongly disagree: 1/101 (1.0%); Disagree: 16/101 (15.8%); Neutral: 14/101 (13.9%); Agree: 49/101 (48.5%); Strongly agree: 21/101 (20.8%).

Likert score (1–5): median (IQR) 4 (3–4) (excluding “I do not know”).

Sex comparison (female vs male; Mann–Whitney U): U = 1055.0; p = 0.815886; FDR q = 0.815886; rank-biserial r = 0.0276.

Years-in-practice comparison (Kruskal–Wallis): H = 0.307; p = 0.958632; FDR q = 0.958632; ε^2 = -0.028.

S2.1.2 Pain-intensity scenarios (Q11–Q20)

Q11. In your opinion, during normal/physiological calving, cows experience pain rated as (0–10).

Descriptive: n = 101; median (IQR) 6 (5–8); mean (SD) 6.2 (1.7).

Severe pain (≥8/10): 27/101 (26.7%). Rating 10/10: 2/101 (2.0%).

Sex comparison (female vs male; Mann–Whitney U): U = 1079.5; p = 0.970028; FDR q = 0.996995; rank-biserial r = 0.0051.

Years-in-practice comparison (Kruskal–Wallis): H = 2.594; p = 0.458559; FDR q = 0.560461; ε^2 = -0.004.

Q12. In your opinion, during difficult calving requiring intervention, cows experience pain rated as (0–10).

Descriptive: n = 101; median (IQR) 9 (9–10); mean (SD) 8.9 (1.5).

Severe pain (≥8/10): 88/101 (87.1%). Rating 10/10: 42/101 (41.6%).

Sex comparison (female vs male; Mann–Whitney U): U = 848.0; p = 0.064624; FDR q = 0.618202; rank-biserial r = 0.2184.

Years-in-practice comparison (Kruskal–Wallis): H = 3.955; p = 0.266379; FDR q = 0.488361; ε^2 = 0.010.

Q13. In your opinion, during mastitis, cows experience pain rated as (0–10).

Descriptive: n = 101; median (IQR) 7 (7–8); mean (SD) 7.4 (1.5).

Severe pain (≥8/10): 50/101 (49.5%). Rating 10/10: 10/101 (9.9%).

Sex comparison (female vs male; Mann–Whitney U): U = 1084.0; p = 0.996995; FDR q = 0.996995; rank-biserial r = 0.0009.

Years-in-practice comparison (Kruskal–Wallis): H = 9.923; p = 0.019236; FDR q = 0.211592; ε^2 = 0.071.

Q14. In your opinion, during recumbency, cows experience pain rated as (0–10).

Descriptive: $n = 101$; median (IQR) 5 (5–7); mean (SD) 5.5 (2.0).

Severe pain ($\geq 8/10$): 16/101 (15.8%). Rating 10/10: 2/101 (2.0%).

Sex comparison (female vs male; Mann–Whitney U): $U = 893.0$; $p = 0.151288$; FDR $q = 0.618202$; rank-biserial $r = 0.1770$.

Years-in-practice comparison (Kruskal–Wallis): $H = 3.193$; $p = 0.362771$; FDR $q = 0.560461$; $\varepsilon^2 = 0.002$.

Q15. In your opinion, during lameness (minimal weight-bearing), cattle experience pain rated as (0–10).

Descriptive: $n = 101$; median (IQR) 7 (6–8); mean (SD) 7.1 (1.7).

Severe pain ($\geq 8/10$): 45/101 (44.6%). Rating 10/10: 11/101 (10.9%).

Sex comparison (female vs male; Mann–Whitney U): $U = 933.0$; $p = 0.257249$; FDR $q = 0.618202$; rank-biserial $r = 0.1401$.

Years-in-practice comparison (Kruskal–Wallis): $H = 7.268$; $p = 0.063823$; FDR $q = 0.351029$; $\varepsilon^2 = 0.044$.

Q16. In your opinion, during metritis, cows experience pain rated as (0–10).

Descriptive: $n = 101$; median (IQR) 6 (5–7); mean (SD) 5.6 (2.0).

Severe pain ($\geq 8/10$): 18/101 (17.8%). Rating 10/10: 2/101 (2.0%).

Sex comparison (female vs male; Mann–Whitney U): $U = 999.5$; $p = 0.526162$; FDR $q = 0.723473$; rank-biserial $r = 0.0788$.

Years-in-practice comparison (Kruskal–Wallis): $H = 5.426$; $p = 0.143115$; FDR $q = 0.487537$; $\varepsilon^2 = 0.025$.

Q17. In your opinion, during respiratory disease, cattle experience pain rated as (0–10).

Descriptive: $n = 101$; median (IQR) 5 (4–6); mean (SD) 4.9 (1.8).

Severe pain ($\geq 8/10$): 9/101 (8.9%). Rating 10/10: 0/101 (0.0%).

Sex comparison (female vs male; Mann–Whitney U): $U = 956.5$; $p = 0.336397$; FDR $q = 0.618202$; rank-biserial $r = 0.1184$.

Years-in-practice comparison (Kruskal–Wallis): $H = 0.821$; $p = 0.844483$; FDR $q = 0.844483$; $\varepsilon^2 = -0.022$.

Q18. In your opinion, during digestive disease, cattle experience pain rated as (0–10).

Descriptive: n = 101; median (IQR) 7 (5–8); mean (SD) 6.6 (1.9).

Severe pain (≥8/10): 33/101 (32.7%). Rating 10/10: 7/101 (6.9%).

Sex comparison (female vs male; Mann–Whitney U): $U = 980.0$; $p = 0.436122$; FDR $q = 0.685334$; rank-biserial $r = 0.0968$.

Years-in-practice comparison (Kruskal–Wallis): $H = 1.493$; $p = 0.683974$; FDR $q = 0.752372$; $\varepsilon^2 = -0.016$.

Q19. In your opinion, during thermal disbudding, calves experience pain rated as (0–10).

Descriptive: n = 101; median (IQR) 8 (7–9); mean (SD) 7.9 (1.9).

Severe pain (≥8/10): 69/101 (68.3%). Rating 10/10: 22/101 (21.8%).

Sex comparison (female vs male; Mann–Whitney U): $U = 1069.5$; $p = 0.910221$; FDR $q = 0.996995$; rank-biserial $r = 0.0143$.

Years-in-practice comparison (Kruskal–Wallis): $H = 4.861$; $p = 0.182284$; FDR $q = 0.487537$; $\varepsilon^2 = 0.019$.

Q20. In your opinion, during castration, bulls experience pain rated as (0–10).

Descriptive: n = 101; median (IQR) 8 (6–9); mean (SD) 7.4 (2.2).

Severe pain (≥8/10): 60/101 (59.4%). Rating 10/10: 21/101 (20.8%).

Sex comparison (female vs male; Mann–Whitney U): $U = 956.5$; $p = 0.337201$; FDR $q = 0.618202$; rank-biserial $r = 0.1184$.

Years-in-practice comparison (Kruskal–Wallis): $H = 4.398$; $p = 0.221608$; FDR $q = 0.487537$; $\varepsilon^2 = 0.014$.

Internal consistency of the 10 cattle pain-scenario ratings was acceptable (Cronbach's $\alpha = 0.747$).

Respondent-level mean pain score across the 10 cattle scenarios: median (IQR) 6.9 (6.1–7.5); mean (SD) 6.8 (1.0).

S2.1.3 Logistic regression: routine discussion of pain with producers

Outcome definition: Q10 coded as 1 for “agree” or “strongly agree” and 0 for “neutral”, “disagree”, or “strongly disagree”; “I do not know” was treated as missing (not observed for Q10). Predictors: sex, years in practice (categorical), emergency medical responder/volunteer firefighter status, and attitudinal items Q4–Q9 entered as 1-point Likert increments.

Cattle model: n = 98 complete cases; likelihood ratio test p = 0.937330; McFadden pseudo R^2 = 0.041.

C(years_cat)[T.6-10]: OR 0.99 (95% CI 0.27–3.53); p = 0.982245.

C(years_cat)[T.11-19]: OR 0.76 (95% CI 0.21–2.75); p = 0.675900.

C(years_cat)[T.20 i więcej]: OR 0.42 (95% CI 0.06–2.90); p = 0.381581.

sex_female: OR 0.47 (95% CI 0.14–1.51); p = 0.204943.

first_responder: OR 0.81 (95% CI 0.11–6.08); p = 0.837155.

q4: OR 0.90 (95% CI 0.56–1.46); p = 0.679194.

q5: OR 1.25 (95% CI 0.78–1.99); p = 0.356774.

q6: OR 0.95 (95% CI 0.62–1.48); p = 0.835790.

q7: OR 1.12 (95% CI 0.71–1.75); p = 0.630375.

q8: OR 0.83 (95% CI 0.53–1.28); p = 0.396599.

q9: OR 1.95 (95% CI 0.74–5.13); p = 0.174372.

S2.2 Pig survey

Demographics

Total responses analyzed: 101.

Sex: 67/101 male (66.3%), 34/101 female (33.7%).

Years in practice: 25/101 (24.8%) 1-5 years; 26/101 (25.7%) 6-10 years; 32/101 (31.7%) 11-19 years; 18/101 (17.8%) ≥20 years.

Emergency medical responder/volunteer firefighter: 4/101 (4.0%).

S2.2.1 Attitudinal items (Q4–Q10)

Q4. It is difficult to recognize pain in pigs.

Strongly disagree: 10/101 (9.9%); Disagree: 56/101 (55.4%); Neutral: 11/101 (10.9%); Agree: 16/101 (15.8%); Strongly agree: 7/101 (6.9%). "I do not know": 1/101 (1.0%).

Likert score (1–5): median (IQR) 2 (2–3) (excluding "I do not know").

Sex comparison (female vs male; Mann–Whitney U): $U = 1106.5$; $p = 0.903927$; FDR $q = 0.903927$; rank-biserial $r = 0.0138$.

Years-in-practice comparison (Kruskal–Wallis): $H = 1.635$; $p = 0.651442$; FDR $q = 0.760016$; $\varepsilon^2 = -0.014$.

Q5. I feel that I know enough about pain and its treatment in pigs.

Strongly disagree: 4/101 (4.0%); Disagree: 20/101 (19.8%); Neutral: 32/101 (31.7%); Agree: 39/101 (38.6%); Strongly agree: 6/101 (5.9%).

Likert score (1–5): median (IQR) 3 (3–4) (excluding "I do not know").

Sex comparison (female vs male; Mann–Whitney U): $U = 1054.5$; $p = 0.525186$; FDR $q = 0.644967$; rank-biserial $r = 0.0742$.

Years-in-practice comparison (Kruskal–Wallis): $H = 3.440$; $p = 0.328677$; FDR $q = 0.575184$; $\varepsilon^2 = 0.005$.

Q6. Pigs are not as sensitive to pain as humans.

Strongly disagree: 24/101 (23.8%); Disagree: 34/101 (33.7%); Neutral: 25/101 (24.8%); Agree: 10/101 (9.9%); Strongly agree: 4/101 (4.0%). "I do not know": 4/101 (4.0%).

Likert score (1–5): median (IQR) 2 (2–3) (excluding "I do not know").

Sex comparison (female vs male; Mann–Whitney U): $U = 696.0$; $p = 0.004395$; FDR $q = 0.030766$; rank-biserial $r = 0.3409$.

Years-in-practice comparison (Kruskal–Wallis): $H = 4.767$; $p = 0.189681$; FDR $q = 0.442589$; $\varepsilon^2 = 0.019$.

Q7. There are not enough analgesic drugs that can be used in pigs with painful conditions.

Strongly disagree: 6/101 (5.9%); Disagree: 43/101 (42.6%); Neutral: 9/101 (8.9%); Agree: 28/101 (27.7%); Strongly agree: 15/101 (14.9%).

Likert score (1–5): median (IQR) 3 (2–4) (excluding “I do not know”).

Sex comparison (female vs male; Mann–Whitney U): $U = 1008.0$; $p = 0.322184$; FDR $q = 0.644967$; rank-biserial $r = 0.1150$.

Years-in-practice comparison (Kruskal–Wallis): $H = 13.210$; $p = 0.004203$; FDR $q = 0.029423$; $\varepsilon^2 = 0.105$.

Q8. Analgesic drugs are too expensive for pig producers to use routinely.

Strongly disagree: 17/101 (16.8%); Disagree: 44/101 (43.6%); Neutral: 8/101 (7.9%); Agree: 22/101 (21.8%); Strongly agree: 7/101 (6.9%). “I do not know”: 3/101 (3.0%).

Likert score (1–5): median (IQR) 2 (2–4) (excluding “I do not know”).

Sex comparison (female vs male; Mann–Whitney U): $U = 981.5$; $p = 0.552829$; FDR $q = 0.644967$; rank-biserial $r = 0.0705$.

Years-in-practice comparison (Kruskal–Wallis): $H = 6.048$; $p = 0.109279$; FDR $q = 0.382476$; $\varepsilon^2 = 0.032$.

Q9. Pigs recover better when pain is mitigated.

Strongly disagree: 0/101 (0.0%); Disagree: 0/101 (0.0%); Neutral: 6/101 (5.9%); Agree: 44/101 (43.6%); Strongly agree: 51/101 (50.5%).

Likert score (1–5): median (IQR) 5 (4–5) (excluding “I do not know”).

Sex comparison (female vs male; Mann–Whitney U): $U = 1061.5$; $p = 0.533160$; FDR $q = 0.644967$; rank-biserial $r = 0.0680$.

Years-in-practice comparison (Kruskal–Wallis): $H = 2.665$; $p = 0.446284$; FDR $q = 0.624797$; $\varepsilon^2 = -0.003$.

Q10. I routinely discuss pain and pain-mitigation options with pig producers.

Strongly disagree: 2/101 (2.0%); Disagree: 30/101 (29.7%); Neutral: 5/101 (5.0%); Agree: 52/101 (51.5%); Strongly agree: 12/101 (11.9%).

Likert score (1–5): median (IQR) 4 (2–4) (excluding “I do not know”).

Sex comparison (female vs male; Mann–Whitney U): $U = 858.5$; $p = 0.027717$; FDR $q = 0.097009$; rank-biserial $r = 0.2463$.

Years-in-practice comparison (Kruskal–Wallis): $H = 0.937$; $p = 0.816446$; FDR $q = 0.816446$; $\varepsilon^2 = -0.021$.

S2.2.2 Pain-intensity scenarios (Q11–Q19)

Q11. In your opinion, during normal/physiological farrowing, sows experience pain rated as (0–10).

Descriptive: n = 101; median (IQR) 6 (5–8); mean (SD) 6.2 (2.0).

Severe pain (≥8/10): 31/101 (30.7%). Rating 10/10: 8/101 (7.9%).

Sex comparison (female vs male; Mann–Whitney U): U = 1015.0; p = 0.369426; FDR q = 0.711447; rank-biserial r = 0.1089.

Years-in-practice comparison (Kruskal–Wallis): H = 2.709; p = 0.438724; FDR q = 0.548405; ε^2 = -0.003.

Q12. In your opinion, during difficult farrowing requiring internal examination, sows experience pain rated as (0–10).

Descriptive: n = 101; median (IQR) 8 (7–10); mean (SD) 8.2 (1.7).

Severe pain (≥8/10): 72/101 (71.3%). Rating 10/10: 30/101 (29.7%).

Sex comparison (female vs male; Mann–Whitney U): U = 959.5; p = 0.187336; FDR q = 0.711447; rank-biserial r = 0.1576.

Years-in-practice comparison (Kruskal–Wallis): H = 0.506; p = 0.917565; FDR q = 0.968007; ε^2 = -0.026.

Q13. In your opinion, during infectious mastitis, sows experience pain rated as (0–10).

Descriptive: n = 101; median (IQR) 8 (7–9); mean (SD) 7.6 (1.8).

Severe pain (≥8/10): 62/101 (61.4%). Rating 10/10: 15/101 (14.9%).

Sex comparison (female vs male; Mann–Whitney U): U = 1021.5; p = 0.391050; FDR q = 0.711447; rank-biserial r = 0.1032.

Years-in-practice comparison (Kruskal–Wallis): H = 5.098; p = 0.164768; FDR q = 0.235382; ε^2 = 0.022.

Q14. In your opinion, during limb fracture, pigs experience pain rated as (0–10).

Descriptive: n = 101; median (IQR) 10 (8–10); mean (SD) 8.9 (1.4).

Severe pain (≥8/10): 85/101 (84.2%). Rating 10/10: 51/101 (50.5%).

Sex comparison (female vs male; Mann–Whitney U): $U = 1104.5$; $p = 0.792229$; FDR $q = 0.880255$; rank-biserial $r = 0.0303$.

Years-in-practice comparison (Kruskal–Wallis): $H = 5.659$; $p = 0.129426$; FDR $q = 0.235382$; $\varepsilon^2 = 0.027$.

Q15. In your opinion, during lameness (minimal weight-bearing), pigs experience pain rated as (0–10).

Descriptive: n = 101; median (IQR) 7 (5–8); mean (SD) 6.6 (1.7).

Severe pain (≥8/10): 27/101 (26.7%). Rating 10/10: 8/101 (7.9%).

Sex comparison (female vs male; Mann–Whitney U): $U = 1124.5$; $p = 0.918219$; FDR $q = 0.918219$; rank-biserial $r = 0.0127$.

Years-in-practice comparison (Kruskal–Wallis): $H = 12.007$; $p = 0.007358$; FDR $q = 0.073577$; $\varepsilon^2 = 0.093$.

Q16. In your opinion, during an open shoulder ulcer, pigs experience pain rated as (0–10).

Descriptive: n = 101; median (IQR) 6 (5–8); mean (SD) 6.3 (2.0).

Severe pain (≥8/10): 29/101 (28.7%). Rating 10/10: 8/101 (7.9%).

Sex comparison (female vs male; Mann–Whitney U): $U = 1024.5$; $p = 0.408005$; FDR $q = 0.711447$; rank-biserial $r = 0.1005$.

Years-in-practice comparison (Kruskal–Wallis): $H = 7.467$; $p = 0.058416$; FDR $q = 0.194721$; $\varepsilon^2 = 0.046$.

Q17. In your opinion, during respiratory disease, pigs experience pain rated as (0–10).

Descriptive: n = 101; median (IQR) 5 (4–7); mean (SD) 5.3 (2.2).

Severe pain (≥8/10): 13/101 (12.9%). Rating 10/10: 6/101 (5.9%).

Sex comparison (female vs male; Mann–Whitney U): $U = 1065.0$; $p = 0.593001$; FDR $q = 0.741251$; rank-biserial $r = 0.0650$.

Years-in-practice comparison (Kruskal–Wallis): $H = 5.487$; $p = 0.139402$; FDR $q = 0.235382$; $\varepsilon^2 = 0.026$.

Q18. In your opinion, during digestive disease, pigs experience pain rated as (0–10).

Descriptive: $n = 101$; median (IQR) 6 (4–7); mean (SD) 5.8 (2.2).

Severe pain (≥8/10): 24/101 (23.8%). Rating 10/10: 8/101 (7.9%).

Sex comparison (female vs male; Mann–Whitney U): $U = 1047.0$; $p = 0.506850$; FDR $q = 0.724072$; rank-biserial $r = 0.0808$.

Years-in-practice comparison (Kruskal–Wallis): $H = 7.978$; $p = 0.046473$; FDR $q = 0.194721$; $\varepsilon^2 = 0.051$.

Q19. In your opinion, during tail docking, piglets experience pain rated as (0–10).

Descriptive: $n = 101$; median (IQR) 5 (3–8); mean (SD) 5.6 (2.7).

Severe pain (≥8/10): 27/101 (26.7%). Rating 10/10: 10/101 (9.9%).

Sex comparison (female vs male; Mann–Whitney U): $U = 976.0$; $p = 0.239534$; FDR $q = 0.711447$; rank-biserial $r = 0.1431$.

Years-in-practice comparison (Kruskal–Wallis): $H = 0.256$; $p = 0.968007$; FDR $q = 0.968007$; $\varepsilon^2 = -0.028$.

Internal consistency of the 9 pig pain-scenario ratings was good (Cronbach's $\alpha = 0.829$).

Respondent-level mean pain score across the 9 pig scenarios: median (IQR) 6.8 (5.9–7.7); mean (SD) 6.7 (1.3).

S2.2.3 Spearman correlations with respondent-level mean pain score

Spearman rank correlations were computed between each attitudinal item (Q4–Q10; numeric scoring excluding "I do not know") and the respondent-level mean pain score. Benjamini–Hochberg FDR adjustment was applied across the 7 correlations.

It is difficult to recognize pain in pigs. (Q4): $\rho = -0.0822$; $p = 0.416102$; FDR $q = 0.416102$; $n = 100$.

I feel that I know enough about pain and its treatment in pigs. (Q5): $\rho = 0.1363$; $p = 0.174063$; FDR $q = 0.203073$; $n = 101$.

Pigs are not as sensitive to pain as humans. (Q6): $\rho = -0.2979$; $p = 0.003042$; FDR $q = 0.021295$; $n = 97$.

There are not enough analgesic drugs that can be used in pigs with painful conditions. (Q7): $\rho = 0.1605$; $p = 0.108750$; FDR $q = 0.172023$; $n = 101$.

Analgesic drugs are too expensive for pig producers to use routinely. (Q8): $\rho = 0.1569$; $p = 0.122874$; FDR $q = 0.172023$; $n = 98$.

Pigs recover better when pain is mitigated. (Q9): $\rho = 0.2029$; $p = 0.041812$; FDR $q = 0.097561$; $n = 101$.

I routinely discuss pain and pain-mitigation options with pig producers. (Q10): $\rho = 0.2086$; $p = 0.036269$; FDR $q = 0.097561$; $n = 101$.

S2.2.4 Logistic regression: routine discussion of pain with producers

Outcome definition and predictors were identical to the cattle analysis (see S2.1.3).

Pig model: n = 93 complete cases; likelihood ratio test p = 0.003537; McFadden pseudo R^2 = 0.234; in-sample AUC = 0.805.

C(years_cat)[T.6-10]: OR 0.43 (95% CI 0.09–2.11); p = 0.298687.

C(years_cat)[T.11-19]: OR 0.34 (95% CI 0.07–1.56); p = 0.163378.

C(years_cat)[T.20 i więcej]: OR 0.34 (95% CI 0.05–2.21); p = 0.259454.

sex_female: OR 3.20 (95% CI 0.86–11.86); p = 0.082104.

first_responder: OR 0.06 (95% CI 0.00–1.10); p = 0.057725.

q4: OR 1.16 (95% CI 0.66–2.03); p = 0.607984.

q5: OR 2.11 (95% CI 1.13–3.93); p = 0.019511.

q6: OR 0.82 (95% CI 0.48–1.42); p = 0.478098.

q7: OR 2.24 (95% CI 1.15–4.37); p = 0.017756.

q8: OR 0.59 (95% CI 0.31–1.10); p = 0.094985.

q9: OR 2.37 (95% CI 0.96–5.85); p = 0.060749.
